# One-Step Generation and Purification of Cell-Encapsulated Hydrogel Microsphere With an Easily Assembled Microfluidic Device

**DOI:** 10.3389/fbioe.2021.816089

**Published:** 2022-01-28

**Authors:** Tao Zhang, Hong Zhang, Wuping Zhou, Keming Jiang, Cong Liu, Ru Wang, Yuanshuai Zhou, Zhiqiang Zhang, Qian Mei, Wen-Fei Dong, Minxuan Sun, Haiwen Li

**Affiliations:** ^1^ School of Biomedical Engineering (Suzhou), Division of Life Sciences and Sciences and Medicine, University of Science and Technology of China, Hefei, China; ^2^ CAS Key Lab of Bio-Medical Diagnostics, Suzhou Institute of Biomedical Engineering and Technology, Chinese Academy of Science, Suzhou, China; ^3^ Jiangsu Key Laboratory of Medical Optics, Suzhou Institute of Biomedical Engineering and Technology, Chinese Academy of Sciences, Suzhou, China; ^4^ School of Life Sciences, Shanghai University, Shanghai, China; ^5^ School of Life Science and Technology, Changchun University of Science and Technology, Changchun, China

**Keywords:** one-step purification, cell-encapsulated, hydrogel microsphere, microfluidic, spheroid

## Abstract

Cell-laden hydrogel microspheres with uniform size show great potential for tissue repair and drug screening applications. Droplet microfluidic systems have been widely used for the generation of cell-laden hydrogel microspheres. However, existing droplet microfluidic systems are mostly based on complex chips and are not compatible with well culture plates. Moreover, microspheres produced by droplet microfluidics need demulsification and purification from oil, which requires time and effort and may compromise cell viability. Herein, we present a simple one-step approach for producing and purifying hydrogel microspheres with an easily assembled microfluidic device. Droplets were generated and solidified in the device tubing. The obtained hydrogel microspheres were then transferred to a tissue culture plate filled with cell culture media and demulsified through evaporation of the oil at 37°C. The removal of oil caused the gelled microspheres to be released into the cell culture media. The encapsulated cells demonstrated good viability and grew into tumor spheroids in 12–14 days. Single cell-laden hydrogel microspheres were also obtained and grown into spheroid in 14 days. This one-step microsphere generation method shows good potential for applications in automated spheroid and organoid cultures as well as drug screening, and could potentially offer benefits for translation of cell/microgel technologies.

**GRAPHICAL ABSTRACT:**
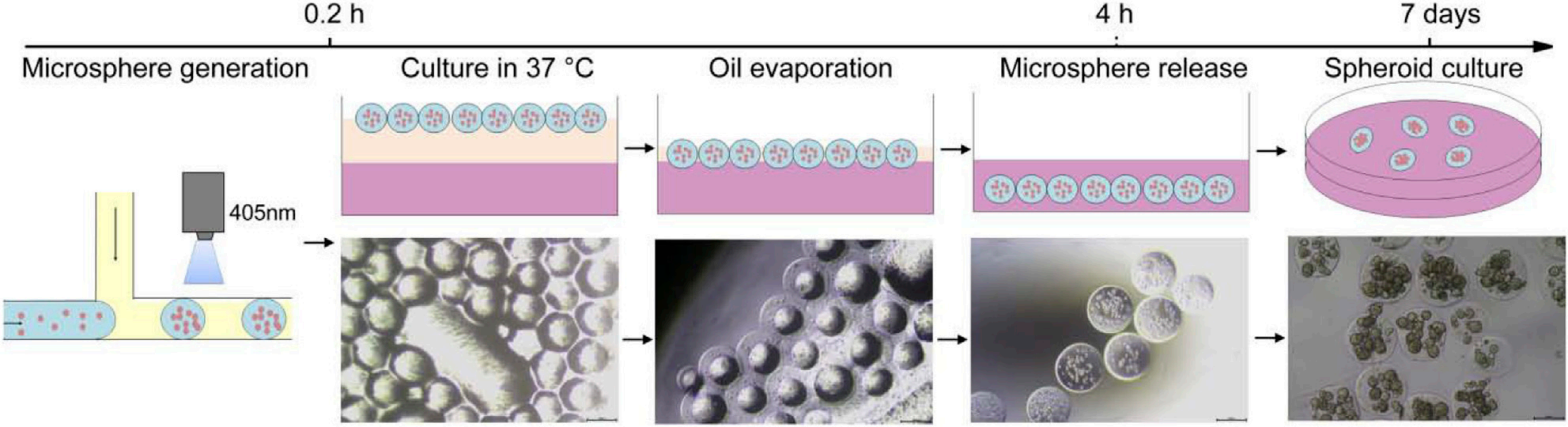


## Introduction

Spheroids are three-dimensional (3D) spherical cellular aggregates that allow cells to interact with neighboring cells and their extracellular matrix. Spheroids can recapitulate cell morphology as well as reproduce gene expression, cell connectivity, and tissue morphology. Compared with two-dimensional models, spheroids are more physiologically relevant and predictive (Du et al., 2008; Dyson, 2019). Spheroids have therefore become the most common and versatile three-dimensional model since they were first used in cell cultures in the 1950s (Dyson, 2019; Boucherit et al., 2020; Sivakumar et al., 2020; Decarli et al., 2021; Roper et al., 2021).

Various scaffold-free and scaffold-based approaches have been developed to generate three-dimensional spheroids. Scaffold-free approaches include using hanging drop plates (Kelm et al., 2003; Guo et al., 2014), low cell attachment plates (Ivanov et al., 2015), and emerging techniques such as magnetic levitation (Türker et al., 2018), ultrasound waves, and acoustic waves. Scaffold-free approaches are easy to perform and can be applied to various cell types (Franchi-Mendes et al., 2021). Microfabrication techniques such as photolithography (Du et al., 2008; Brandenberg et al., 2020) and micromolding (Tekin et al., 2011; Tao et al., 2019) have also been used to create spheroids, allowing the volume and shape of spheroids to be precisely controlled. However, most spheroid fabrication approaches require an excessive amount of time and effort, and producing uniform spheroids with a high throughput and in a reproducible manner is particularly challenging.

Droplet microfluidics is a versatile technology that allows for the manipulation of small volumes of liquids on a microfluidic chip (Zhu et al., 2019; Mohamed et al., 2020; Balasubramanian et al., 2021). Previous studies have applied droplet microfluidics in the fields of molecular and cellular biology and analytical chemistry for applications such as clonal development (Dolega et al., 2015), drug screening (Courtney et al., 2017), single-molecule/single-cell analysis (Najah et al., 2012; Shembekar et al., 2018; Shao et al., 2020), tissue engineering (Liu et al., 2018), organoid modeling (Vadivelu et al., 2017; Zhang et al., 2021), and spheroid fabrication (Lopa et al., 2020; Mohamed et al., 2020). Droplet microfluidic technology is especially suitable for 3D cell cultures (Eqbal et al., 2021). Three-dimensional cell culture technology based on microfluidic droplets has many advantages. For instance, droplet size distributions from tens to hundreds of microns allow nutrients and oxygen to effectively diffuse into cells. In addition, droplet microfluidic systems provide high throughput and high controllability, and they can produce uniform cell-encapsulated microspheres with tunable microenvironments in a high throughput manner. Currently, various materials and microfluidic chips have been used to generate hydrogel microspheres for cell culture and spheroid fabrication.

The preparation of microdroplets by microfluidic methods typically uses two incompatible liquids as a continuous phase and a discrete phase, and the generation of droplets is controlled by the microtubule structure and two-phase flow rate ratio of the microfluidic chip. Through precise pressure control, uniform and high-throughput droplet generation can be achieved. The obtained droplets can then be solidified into microspheres by a photosensitive or thermal reaction. After gelation, demulsification and centrifugation are carried out to obtain the encapsulated hydrogel microspheres (Caballero Aguilar et al., 2021). However, this method is complex and time-consuming, and exposing the cells to demulsifiers affects their viability. Alternatively, other studies have used an aqueous two-phase system as a template for hydrogel microspheres generation in water to avoid extra washing steps (Moon et al., 2015; Mao et al., 2017; Liu et al., 2018). However, aqueous two-phase systems require two water phase reagents that are immiscible with each other. Only a limited number of reagent combinations, such as polyethylene glycol and dextran, meet this requirement. Therefore, the limited number of solution combinations hinders the wide application of this technology in cell encapsulation.

To avoid this problem, Choi et al. developed a method for the one-step generation of monodisperse cell-laden hydrogel microspheres. They first generated double emulsion droplets with an ultrathin oil shell, then used UV light to solidify the droplets. After polymerization of the inner droplets, the thin oil shells of the double emulsion droplets gradually wet and subsequently transfer into the aqueous solution. This results in hydrogel microspheres dispersed in the aqueous phase without extra washing steps (Choi et al., 2016). However, this method requires complex microfluidic control and is not compatible with conventional cell culture plate systems. Recently, on-chip methods for the generation and purification of hydrogel microspheres in an integrated microfluidic chip have been developed (Deng et al., 2011; Hong et al., 2012; Choi et al., 2016; Mohamed et al., 2019). Deng et al. designed filter blocks in a microfluidic chip for on-chip hydrogel microsphere filtration. The filter blocks separated hydrogel microspheres from an oil phase by infusing the oil phase and the aqueous phase. After filtration, the hydrogel microspheres were sequentially maintained by the filter blocks (Deng et al., 2011). In another study, Hong et al. extracted collagen microspheres from a mineral oil phase into a cell culture with an aqueous extraction chamber. The fluid exchange and filter gates on the chip aided the separation and release of the microspheres into the aqueous solution (Hong et al., 2012). Other microfluidic chip-integrated on-chip fabrication and purification methods and off-microfluidic approaches have also been used for hydrogel microsphere purification (Lee et al., 2014). For instance, hydrogel microspheres have filtered by a micropost array to achieve their removal from an oil phase to an aqueous phase (Mohamed et al., 2019). However, these methods require complex chip design and processing steps.

In this study, a simple one-step approach for generating and purifying encapsulated hydrogel microspheres was designed. Droplets were generated with a commercial adapter and solidified in the system’s tubing. The hydrogel microspheres were then transferred to a tissue culture plate filled with cell culture media. The gelled microspheres were demulsified through oil evaporation, causing them to be released into the cell culture media. The obtained encapsulated cells maintained good viability and grew into tumor spheroids in 14 days. The one-step microsphere generation method presented herein may have potential application in biomedical fields.

## Methods

### Materials

Gelatin methacryloyl (GelMA) and Lithium phenyl(2,4,6-trimethylbenzoyl) phosphinate (LAP) were obtained from Stemeasy Biotech Co., Ltd. (Wuxi, China). HFE7500 fluorinated oil were purchased from 3M Company (Shanghai, China), and SF33 fluorinated oil were purchased from Chemours Co., Ltd. (Shanghai, China). Perfluoropolyether–polyethylene glycol (PFPE–PEG) block-copolymer fluorosurfactants (PEG-based fluorosurfactants) were synthesized as described previously (Holtze et al., 2008). Calcein-AM/PI Double Stain Kit was purchased from Yeasen Biotech Co., Ltd. (Shanghai, China). 0.22 µm filters were purchased from Millipore (Bedford, MA, United States). All other chemicals were analytical grade, and double-distilled water was used throughout the experiments. MicroCross were obtained from IDEX Health & Science LLC (Oak Harbor, WA, United States).

### MicroCross Construction and Droplet Generation

**FIGURE 1:**
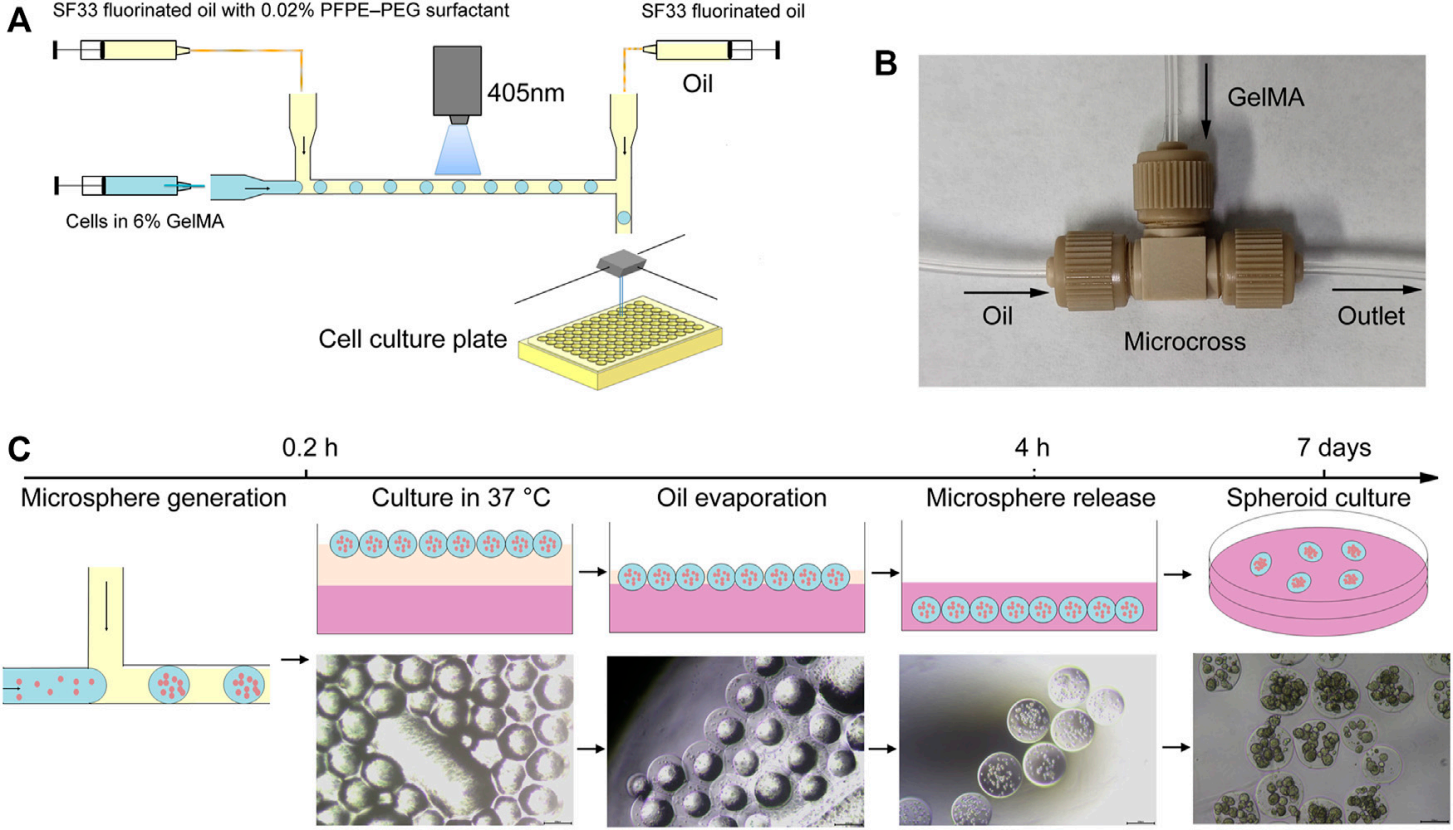
Schematic illustration of the developed microfluidic system for one-step hydrogel microsphere generation. **(A)** Schematic diagram of the microfluidic platform. **(B)** Configuration of the microfluidic chip. **(C)** Generation and separation procedures for obtaining hydrogel microspheres.

A microfluidic T-junction platform was designed and constructed for droplet generation, as shown in Figure 1. An IDEX MicroCross T-junction and a fluorinated ethylene-polypropylene (FEP) tube were used for device fabrication. The inner diameter of the MicroCross was 250 μm. HFE7500 fluorinated oil containing 1% PFPE–PEG fluorinated surfactant was selected as the continuous phase and 6% photocrosslinkable gelatin, GelMA in PBS, containing 0.4% photoinitiator LAP was selected as the disperse phase. To generate droplets, the continuous phase and disperse phase were loaded into two syringes (20 and 1 ml, respectively), which were propelled by peristaltic pumps (Baoding Longer Precision Pump Co., Ltd.). At the junction section, the disperse phase was broken into small droplets under the shear force caused by the continuous phase.

### Generation of Cell-Laden Hydrogel Microspheres

HCT116 and U87 cells were digested by trypsin-EDTA and resuspended in DMEM supplemented with 10% FBS at a density of 8×10^6^ cells/ml. Photocrosslinkable GelMA and photoinitiator LAP were dissolved in PBS and filtered with a 0.22 µm filter. The disperse phase was prepared by mixing 100 μl cell suspension with 200 µL 12% GelMA and 100 µL 1.6% LAP. A volatile fluorinated oil SF33 with low boiling point (33°C) containing 0.02% PFPE–PEG fluorosurfactant was chosen as the continuous phase. The continuous phase and disperse phase were loaded into two syringes (20 and 1 ml, respectively), which were propelled by peristaltic pumps. Droplets generated at the T-junction were cured using 405 nm light for 15 s. The obtained hydrogel microspheres were transferred to a tissue culture plate filled with cell culture media by an second oil syringe with SF33 fluorinated oil downstream of emulsification. Cells were cultured at 37°C in a humidified 5% CO_2_ and 95% air atmosphere.

### Cell Viability Assessment

Cell viability was assessed using a commercial Calcein-AM/PI Double Stain Kit (Shanghai Yeasen Biotech. Co., Ltd.) according to the manufacturer’s instructions. Live/dead staining solution was prepared by adding 1 µl calcein AM and 3 µL ethidium homodimer into 1 ml assay buffer. The cell-laden hydrogel microspheres were harvested and centrifuged at 1,000 rpm for 5 min. Then, the cell-laden hydrogel microspheres were suspended in assay buffer and stained by live/dead staining solution at room temperature for 20 min. After staining, the hydrogel microspheres were imaged using a fluorescence microscope. The live and dead cell numbers were identified and the ratio of live cells to the total number of cells was determined using ImageJ software (Ma et al., 2017).

### Statistical Analysis

Each experiment was performed at least three times. Data were presented as mean± standard deviation. Image analysis, data treatments, and statistical analysis were performed using ImageJ and Origin. Statistical analysis was performed using one-way ANOVA, and statistical significance was established at *p* < 0.05.

## Results

### Design and Fabrication of the Assembled Microfluidic Platform

To generate and purify cell-encapsulated hydrogel microspheres in one step, we developed an easily assembled microfluidic platform. The microfluidic platform consisted of three major modules: a T-junction microfluidic module for droplets generation, a blue light curing module for on-line hydrogel microsphere gelation, and a microsphere injection module for microsphere distribution and purification (Figure 1A). The T-junction microfluidic module was designed and fabricated using commercial IDEX adapters. To generate droplets of an appropriate volume, several adapters were assessed, and micro static mixing T-junction with 250 μm and 150 internal diameter were chosen for droplet generation (Figure 1B). After droplet generation, the droplets were immediately solidified using a blue light (wavelength of 405 nm, power density of 2000 mW/cm^2^) in the FEP tube (Supplementary Video S1). An second oil syringe downstream of emulsification was used to increase the spacing between droplet and distribute the hydrogel microspheres into cell culture plates containing cell culture media. When the microsphere reaches the outlet, the second oil syringe extruded the microsphere into the 96 well plate, and then the tube outlet moved to the next position through the two-dimensional mobile platform, and extruded the next drop of microsphere into the 96 well plate. The cell culture plates were then transferred to a CO_2_ incubator. In the 37°C environment, the oil phase gradually evaporated within 4 h, leading to the direct transfer of the gel microspheres into the cell culture media without an additional washing step (Figure 1C).

### Formation of Emulsion Droplets With the Assembled Microfluidic System

**FIGURE 2:**
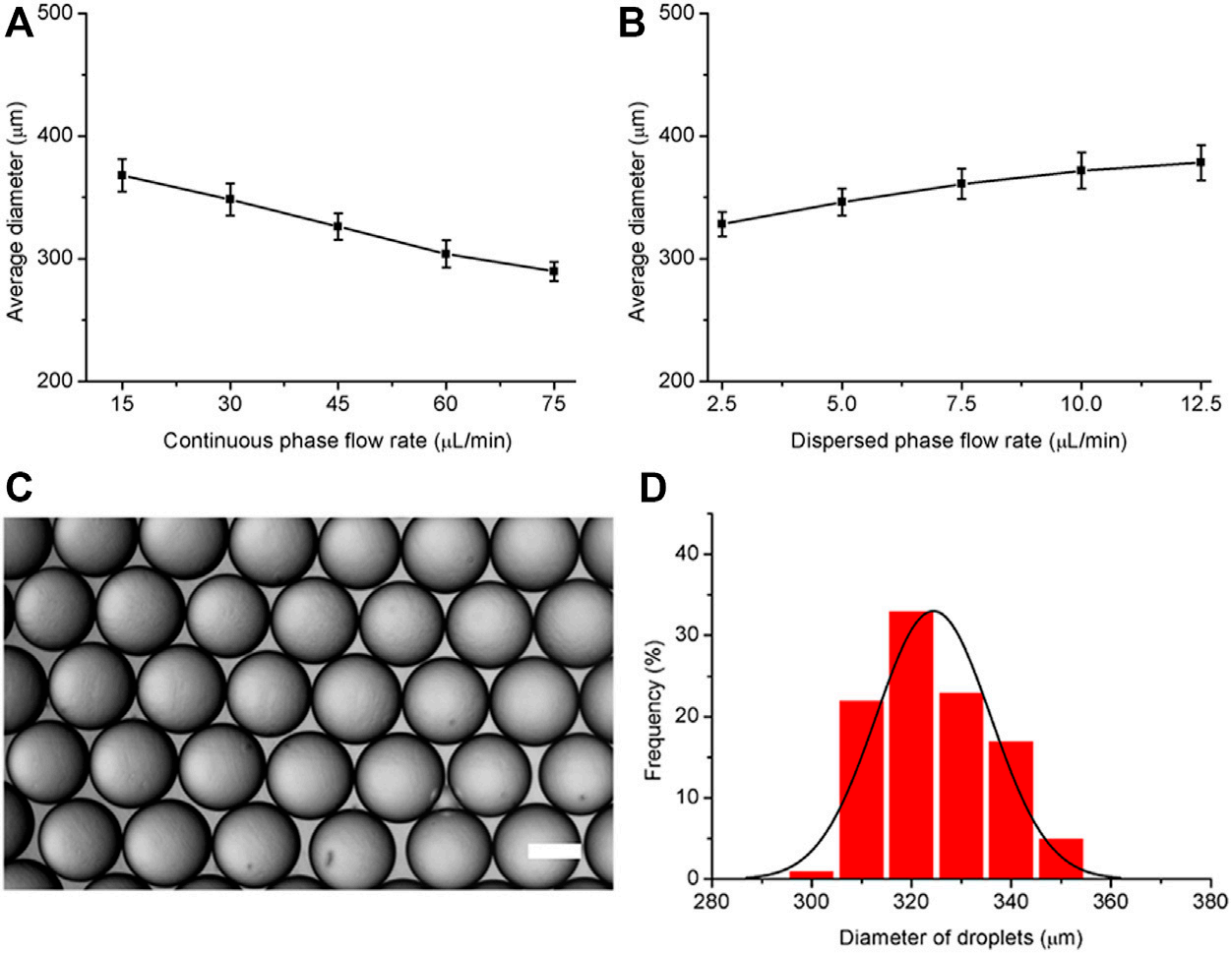
Effects of the disperse phase and continuous phase flow rates on the diameter of generated droplets. **(A)** Variation in droplet diameter with increasing continuous phase flow rate from 15 to 75 μl/min (disperse phase flow rate: 5 μl/min). **(B)** Variation in droplet diameter with increasing disperse phase flow rate from 2.5 to 12.5 μl/min (continuous phase flow rate: 30 μl/min). **(C)** Representative microscopy image of droplets generated by the microfluidic system. **(D)** Size distribution of the droplets. The scale bar represents 200 µm.

To assess the efficiency of the assembled microfluidic system for droplet generation, HFE7500 fluorinated oil and SF33 fluorinated oil were tested. HFE7500 oil is less volatile and the droplets generated are stable at room temperature. In contrast, SF33 oil is volatile and the droplets are not as stable as droplets generated with HFE7500 oil and some droplets fuse when the droplets gather together. So we chose HFE7500 oil for the initial optimization of fabrication conditions. HFE7500 with 1 wt% PFPE–PEG fluorosurfactant and 6% GelMA in PBS were loaded into two syringes and propelled by peristaltic pumps. At the T-junction, the disperse phase was broken into small droplets under the shear force generated by the continuous phase. To evaluate the effect of the continuous phase on droplet generation, the flow rate of the continuous phase was increased from 15 to 75 μl/min while the flow rate of the disperse phase was held constant at 5 μl/min. The results show that the droplet size gradually decreased from 368 to 290 µm as the continuous phase flow rate increased to 75 μl/min (Figure 2A). To test the effect of the disperse phase on droplet generation, the flow rate of the disperse phase was increased from 2.5 to 12.5 μl/min while the flow rate of the continuous phase was held constant at 30 μl/min. The results in Figure 2B show that the droplet size increased when the flow rate of the disperse phase was increased. These results reveal that the droplet size can be adjusted by the flow rates of the continuous phase and the disperse phase. It was also determined that droplets are less likely to collide and coalescence and the droplet size becomes more uniform with increasing flow rate ratio of the continuous phase to the disperse phase. And the results also indicated that the assembled microfluidic system could generate monodispersed emulsion droplets and the average diameter was 326 ± 11 µm (Figures 2C,D).

### Production of Monodisperse Hydrogel Microspheres Through Oil Evaporation

**FIGURE 3:**
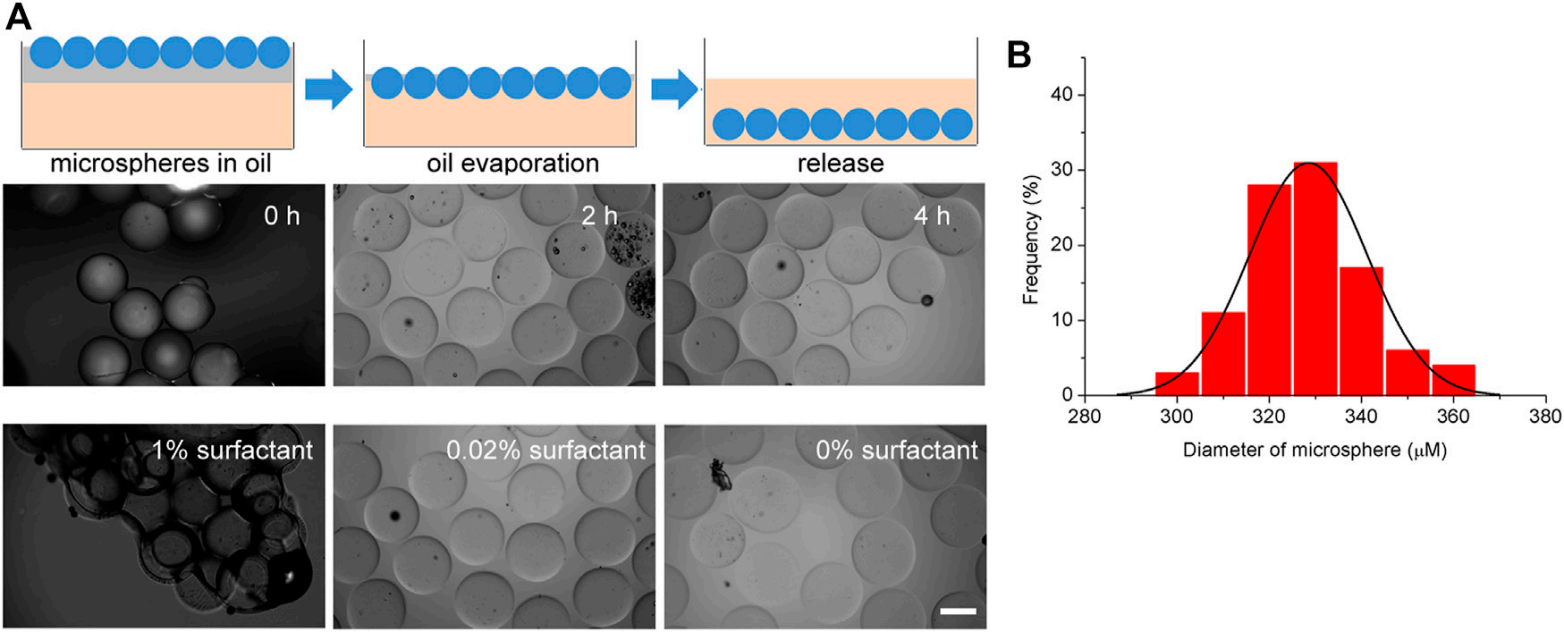
Generation and extraction of hydrogel microspheres. **(A)** Schematic diagram of the microsphere extraction process by oil evaporation. **(B)** Size distribution of the hydrogel microspheres. **(C)** Representative microscopy image of extraction process. The scale bar represents 200 µm.

To produce monodisperse hydrogel microspheres, a volatile fluorinated oil SF33 with low boiling point (33°C) was used. HFE7500 oil was not used because HFE7500 oil is less volatile and the use of HFE7500 is not compatible with the oil evaportation method. Different from the demulsification and purification methods used before, we report a hydrogel microsphere production method without demulsification and washing steps. After the droplets were generated and solidified by 405 nm light for 15 s in the microfluidic system tubing, the resulting hydrogel microspheres were transferred to a cell culture plate filled with cell culture media. Although the density of fluorinated oil SF33 (1.36 g/ml) was higher than that of culture medium, the microspheres in the fluorinated oil remained on top of the cell culture media due to interfacial tension. The tissue culture plate was then placed in a 37°C incubator. Because the incubator temperature was higher than the boiling point of the fluorinated oil (33°C), the oil phase evaporated in 4 h, destroying the stability of the surfactant layer on the droplet surfaces. As a result, the hydrogel microspheres were gradually released from the oil phase into the cell culture medium over 4 h (Figure 3A). The size distribution of the hydrogel microsphere indicated microspheres were monodisperse and the average diameter was 328 ± 13 µm (Figure 3B). It should be noted that the surfactant concentration in the continuous phase should be within an appropriate range. When the surfactant concentration is lower than 0.02%, droplet coalescence occurs before photocuring takes place. When the surfactant concentration is too high, such as 1%, the microspheres aggregate together after oil partially evaporation. Microspheres hang on the surface of the aqueous phase due to the hydrophobic effect of surfactants and are unable to be released into the aqueous phase (Figure 3C).

### Generation of Tumor Spheroid Using the Microfluidic Platform

**FIGURE 4:**
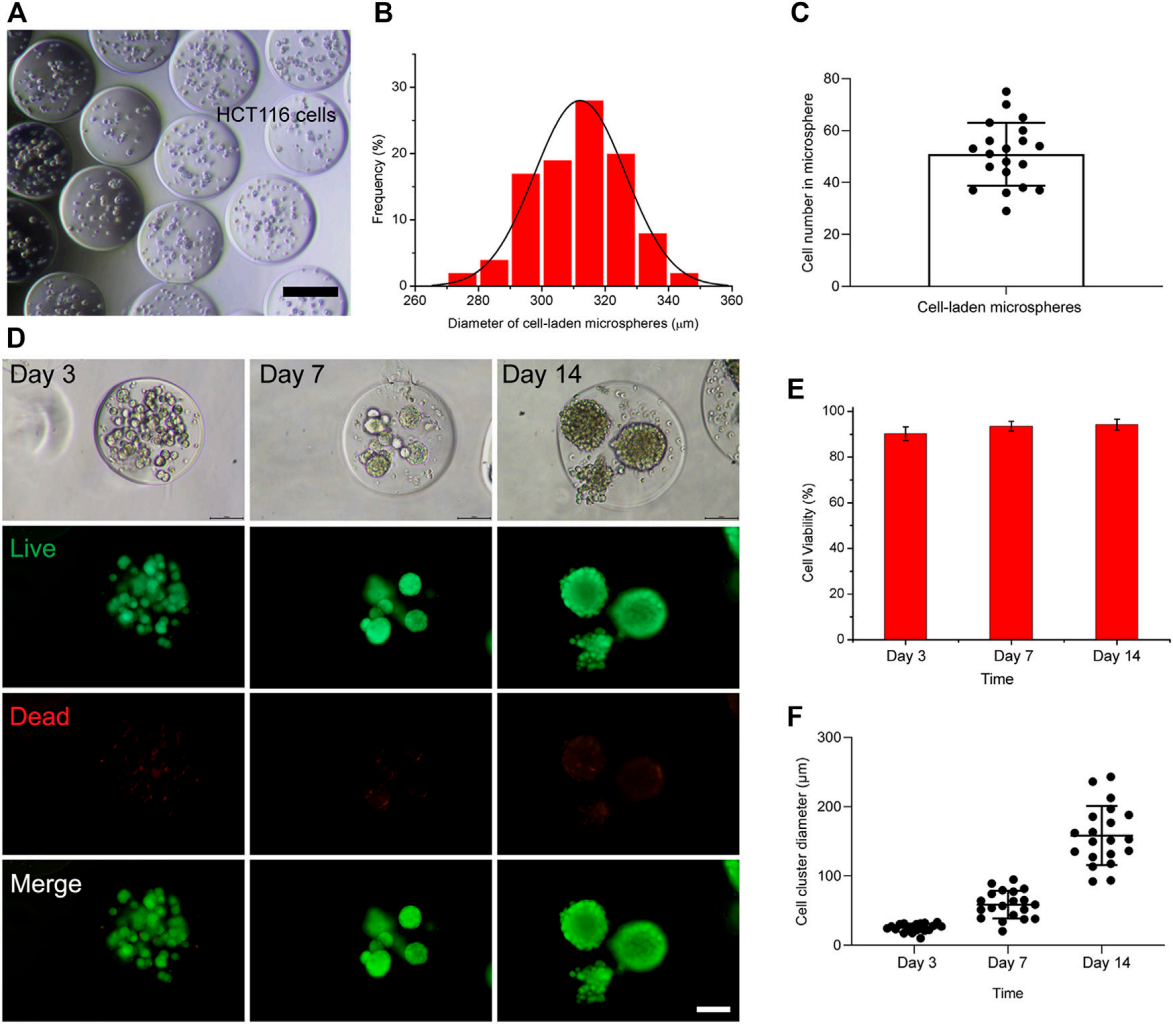
Generation of cell-laden hydrogel microspheres in the microfluidic system. **(A)** Representative image of cell-laden hydrogel microspheres. The scale bar represents 200 µm. **(B)** Size distribution of cell-laden hydrogel microspheres. **(C)** The number of cell-encapsulated in the hydrogel microspheres. **(D)** Fluorescence microscopy image of HCT116-laden hydrogel microspheres after culturing for 3, 7, and 14 days. The scale bar represents 100 µm. **(E)** Percentage viability of encapsulated cells in the hydrogel microspheres of 3, 7, and 14 days. **(F)** The diameter of the cell spheroids formed within the hydrogel microspheres after culturing for 3, 7, and 14 days.

The utility of the hydrogel microsphere production method for the generation of tumor spheroid was studied. HCT116 cells were suspended in 6% GelMA and encapsulated into droplets (Figure 4A). The size distribution of these hydrogel microspheres was then measured. As shown in Figure 4B, the average diameter of the cell-laden hydrogel microspheres was 313 µm within a 5% coefficient of variation, indicating that they were homogeneous. The number of cell-encapsulated in the microspheres was 50 ± 12 µm (Figure 4C). Cell viability was evaluated after encapsulation, and the results show that the HCT116 cells maintained good viability and formed cell clusters in the hydrogel microspheres after 7–14 days of culturing (Figures 4D–F).This microfluidic system was further verified with the U87 tumor cell line. In contrast to the densely packed cellular organization formed by HCT116 cells (Supplementary Figure S1), the U87 cells behaved completely differently after being encapsulated into hydrogel microspheres. The U87 cells spread out from the hydrogel microspheres after culturing for 12 days (Supplementary Figure S2). The cell viability of the encapsulated U87 cells was assessed with a live cell staining kit. The results show that the encapsulated cell viability was comparable to that of non-encapsulated cells, indicating that this microfluidic system is highly biocompatible with 3D cell cultures.

Single cell-laden hydrogel microspheres have been shown to have great potential in the fields of stem cells and tissue engineering. Herein, we generated single U87 tumor cell-laden hydrogel microspheres by adjusting the diameter of the microcross (Supplementary Figure S3A). 100 µm microspheres which is the favorable size for single cell applications were generated. Single cell tumor spheroid formed after 14 days culture, and the live cell staining revealed the U87 cells maintained good viability (Supplementary Figure S3B).

## Discussion

Prior work has documented the potential of droplet microfluidic in spheroid and organoid generation. For example, uniform patient-derived tumor spheroids and engineering human islet organoids have been fabricated using droplet microfluidic based platform (Siltanen et al., 2016; Tao et al., 2019). Droplet microfluidic ensures high-throughput formation of cell-encapsulated hydrogel microspheres of controlled size and cell seeding density by adjusting flow velocity and cell suspension concentration. Morever, droplet microfluidic based platform requires only 30–50% percentage of 3D culture medium compared with traditional 3D culture. Besides, hydrogel microspheres generated by droplet microfluidic can be used combing with other microfluidic device for more complex interdisciplinary studies. However, current technologies for cell-laden microsphere generation need either demulsification and centrifugation or complex microfluidic device to transfer the encapsulated hydrogel microspheres into the culture medium (Caballero Aguilar et al., 2021). These methods are complex and time-consuming, and exposing the cells to demulsifiers affects their viability (Mohamed et al., 2020).

In this study, we developed an easily assembled microfluidic platform for one-step and high-throughput monodisperse hydrogel microsphere preparation and cell encapsulation. Compared with traditional hydrogen microsphere fabrication methods, this method does not require the addition of a demulsifier or other chemicals, reducing the toxic effect on cells and improving the cell survival rate. Evaporation-based microfluidic strategy has been used to produce cell encapsulated hydrogel microsphere. However, evaporation of oil phase resulted in an aggregation of cell encapsulated hydrogel microspheres, and only 40% percentage of cells survive after 6 days culture (Fan et al., 2015). We found that the realization of hydrogel microsphere fabrication without a separate washing step relies on the concentration of surfactant in the oil phase. The surfactant concentration should not be too high, so as to ensure that hydrogel microspheres don’t aggregate and can be released into the culture medium. The surfactant concentration also should not be too low, so as to ensure that the droplets do not fuse before gelation. By adjusting the concentration of surfactant, monodisperse hydrogel microspheres were prepared by real-time solidification in the microfluidic system tube, and over 90% percentage of cells keep alive after 14 days culture. It should be noted that the surfactant in the oil phase should be biocompatible, otherwise the residual surfactant after oil volatilization may affect cell survival (Clausell-Tormos et al., 2008).

Several studies have indicated that spheroids above 200 µm undergo core necrosis (Vadivelu et al., 2017; Corrado et al., 2019). Due to the limitation of mass transport, cells encapsulated in the hydrogel microspheres suffer from different levels of waste products, low nutrients and even hypoxia. Hypoxia may induce cell death in the hydrogel microspheres of a few hundred micrometers (Ling et al., 2007). Previous study indicated that the microsphere diffusivity depends on the microsphere size relative to the network pore size and also on the stress relaxation dynamics of the network. The diffusion of nutrients, growth factors and other molecules in hydrogel can change depending the degree of crosslinking (Burla et al., 2020). Studies also indicated that the concentration of GelMA and its degree of crosslinking are inversely proportional to the degree of porosity and the network pore size, and the decrease of gel concentration is correlated with decreased stiffness of the gel, which promotes cell proliferation and survival (Cuvellier et al., 2021); [45]. Compared with 7.5–10% GelMA used in previous study, low-concentration (6%) GelMA is chosen for cell-encapsulated microsphere generation in our study. By reducing the LAP concentration and the UV exposure time, the viability of the cells are improved while keeping the stability of the microsphere. Moreover, we found that low-concentration gelma microspheres were more likely to aggregate when separated by demulsification and centrifugation, while the microspheres were more likely to disperse rather than aggregate through the oil-phase evaporation and hydrophilic adsorption separation.

Besides, the assembled microfluidic platform is compatible with existing 96 well plates and other cell culture systems, which is conducive to the realization of high-throughput three-dimensional culturing and screening. This method is expected to have important application value in the fields of tumor high-throughput drug screening and organoid preparation.

However, some limitations are worth noting. Cells such as HCT116 generated more than one cluster in one microsphere. Similar results has also been reported in prevrious study (Choi et al., 2016). This may affects the uniform of spheroid generated. Recently, cell aggregation in low-concentration matrigel has been used for uniform spheroids generation. Cells sediment by gravity and aggregate into tumor spheroids (Brandenberg et al., 2020). Cell aggregation for uniform spheroids generation in droplet microfluidic should be further evaluated. It also should be noted that many of the encapsulated cells in the system were located in a non-central position in the hydrogel microspheres, resulting in the partial encapsulation of the cells and allowing some cells to escape into the cell culture. Non-central encapsulation can lead to asymmetric biomechanical stimuli and biochemical culture conditions, which may affect the uniformity of the generated spheroids or organoids. Hence, a strategy for centering the encapsulated cells in the hydrogel microspheres for long-term 3D culturing needs to be developed in the future.

## Data Availability

The original contributions presented in the study are included in the article/Supplementary Material, further inquiries can be directed to the corresponding authors.

## References

[B1] Balasubramanian S. Chen J. Wigneswaran V. Bang-Berthelsen C. H. Jensen P. R. (2021). Droplet-Based Microfluidic High Throughput Screening of Corynebacterium Glutamicum for Efficient Heterologous Protein Production and Secretion. Front. Bioeng. Biotech. 9. 10.3389/fbioe.2021.668513 PMC8137953 34026744

[B2] Boucherit N. Gorvel L. Olive D. (2020). 3D Tumor Models and Their Use for the Testing of Immunotherapies. Front. Immunol. 11. 10.3389/fimmu.2020.603640 PMC7758240 33362787

[B3] Brandenberg N. Hoehnel S. Kuttler F. Homicsko K. Ceroni C. Ringel T. (2020). High-throughput Automated Organoid Culture via Stem-Cell Aggregation in Microcavity Arrays. Nat. Biomed. Eng. 4 (9), 863–874. 10.1038/s41551-020-0565-2 32514094

[B4] Burla F. Sentjabrskaja T. Pletikapic G. van Beugen J. Koenderink G. H. (2020). Particle Diffusion in Extracellular Hydrogels. Soft Matter 16 (5), 1366–1376. 10.1039/c9sm01837a 31939987

[B5] Caballero Aguilar L. M. Duchi S. Onofrillo C. O’Connell C. D. Di Bella C. Moulton S. E. (2021). Formation of Alginate Microspheres Prepared by Optimized Microfluidics Parameters for High Encapsulation of Bioactive Molecules. J. Colloid Interf. Sci. 587, 240–251. 10.1016/j.jcis.2020.12.026 33360897

[B6] Choi C.-H. Wang H. Lee H. Kim J. H. Zhang L. Mao A. (2016). One-step Generation of Cell-Laden Microgels Using Double Emulsion Drops with a Sacrificial Ultra-thin Oil Shell. Lab. Chip 16 (9), 1549–1555. 10.1039/c6lc00261g 27070224 PMC5598084

[B7] Clausell-Tormos J. Lieber D. Baret J.-C. El-Harrak A. Miller O. J. Frenz L. (2008). Droplet-based Microfluidic Platforms for the Encapsulation and Screening of Mammalian Cells and Multicellular Organisms. Chem. Biol. 15 (5), 427–437. 10.1016/j.chembiol.2008.04.004 18482695

[B8] Corrado B. Gregorio V. Imparato G. Attanasio C. Urciuolo F. Netti P. A. (2019). A Three‐dimensional Microfluidized Liver System to Assess Hepatic Drug Metabolism and Hepatotoxicity. Biotechnol. Bioeng. 116 (5), 1152–1163. 10.1002/bit.26902 30552666

[B9] Courtney M. Chen X. Chan S. Mohamed T. Rao P. P. N. Ren C. L. (2017). Droplet Microfluidic System with On-Demand Trapping and Releasing of Droplet for Drug Screening Applications. Anal. Chem. 89 (1), 910–915. 10.1021/acs.analchem.6b04039 27959505

[B10] Cuvellier M. Ezan F. Oliveira H. Rose S. Fricain J.-C. Langouët S. (2021). 3D Culture of HepaRG Cells in GelMa and its Application to Bioprinting of a Multicellular Hepatic Model. Biomaterials 269. 10.1016/j.biomaterials.2020.120611 33385685

[B11] Decarli M. C. Amaral R. Dos Santos D. P. Tofani L. B. Katayama E. Rezende R. A. (2021). Cell Spheroids as a Versatile Research Platform: Formation Mechanisms, High Throughput Production, Characterization and Applications. Biofabrication 13 (3). 10.1088/1758-5090/abe6f2 33592595

[B12] Deng Y. Zhang N. Zhao L. Yu X. Ji X. Liu W. (2011). Rapid Purification of Cell Encapsulated Hydrogel Beads from Oil Phase to Aqueous Phase in a Microfluidic Device. Lab. Chip 11 (23), 4117–4121. 10.1039/c1lc20494g 22012540

[B13] Dolega M. E. Abeille F. Picollet-D'hahan N. Gidrol X. (2015). Controlled 3D Culture in Matrigel Microbeads to Analyze Clonal Acinar Development. Biomaterials 52, 347–357. 10.1016/j.biomaterials.2015.02.042 25818441

[B14] Du Y. Lo E. Ali S. Khademhosseini A. (2008). Directed Assembly of Cell-Laden Microgels for Fabrication of 3D Tissue Constructs. Proc. Natl. Acad. Sci. 105 (28), 9522–9527. 10.1073/pnas.0801866105 18599452 PMC2474514

[B15] Dyson P. J. (2019). The Rise of 3D Cellular Spheroids: Efficient Culture via Upward Growth from a Superamphiphobic Surface. Natl. Sci. Rev. 6 (6), 1068–1069. 10.1093/nsr/nwz158 34691974 PMC8291403

[B16] Eqbal M. D. Naaz F. Sharma K. Gundabala V. (2021). Microfluidics-based Generation of Cell Encapsulated Microbeads in the Presence of Electric fields and Spatio-Temporal Viability Studies. Colloid Surf. B 208, 112065. 10.1016/j.colsurfb.2021.112065 34478958

[B17] Fan R. Naqvi K. Patel K. Sun J. Wan J. (2015). Evaporation-based Microfluidic Production of Oil-free Cell-Containing Hydrogel Particles. Biomicrofluidics 9 (5), 052602. 10.1063/1.4916508 25825624 PMC4376759

[B18] Franchi-Mendes T. Lopes N. Brito C. (2021). Heterotypic Tumor Spheroids in Agitation-Based Cultures: A Scaffold-free Cell Model that Sustains Long-Term Survival of Endothelial Cells. Front. Bioeng. Biotech. 9. 10.3389/fbioe.2021.649949 PMC8219978 34178955

[B19] Guo W. M. Loh X. J. Tan E. Y. Loo J. S. C. Ho V. H. B. (2014). Development of a Magnetic 3D Spheroid Platform with Potential Application for High-Throughput Drug Screening. Mol. Pharmaceutics 11 (7), 2182–2189. 10.1021/mp5000604 24842574

[B20] Holtze C. Rowat A. C. Agresti J. J. Hutchison J. B. Angilè F. E. Schmitz C. H. J. (2008). Biocompatible Surfactants for Water-In-Fluorocarbon Emulsions. Lab. Chip 8 (10), 1632–1639. 10.1039/b806706f 18813384

[B21] Hong S. Hsu H.-J. Kaunas R. Kameoka J. (2012). Collagen Microsphere Production on a Chip. Lab. Chip 12 (18), 3277–3280. 10.1039/c2lc40558j 22824954

[B22] Ivanov D. P. Parker T. L. Walker D. A. Alexander C. Ashford M. B. Gellert P. R. (2015). *In Vitro* co-culture Model of Medulloblastoma and Human Neural Stem Cells for Drug Delivery Assessment. J. Biotechnol. 205, 3–13. 10.1016/j.jbiotec.2015.01.002 25592050

[B23] Kelm J. M. Timmins N. E. Brown C. J. Fussenegger M. Nielsen L. K. (2003). Method for Generation of Homogeneous Multicellular Tumor Spheroids Applicable to a Wide Variety of Cell Types. Biotechnol. Bioeng. 83 (2), 173–180. 10.1002/bit.10655 12768623

[B24] Lee D.-H. Jang M. Park J.-K. (2014). Rapid One-step Purification of Single-Cells Encapsulated in Alginate Microcapsules from Oil to Aqueous Phase Using a Hydrophobic Filter Paper: Implications for Single-Cell Experiments. Biotechnol. J. 9 (10), 1233–1240. 10.1002/biot.201400319 25130499

[B25] Ling Y. Rubin J. Deng Y. Huang C. Demirci U. Karp J. M. (2007). A Cell-Laden Microfluidic Hydrogel. Lab. Chip 7 (6), 756–762. 10.1039/b615486g 17538718

[B26] Liu H. T. Wang H. Wei W. B. Liu H. Jiang L. Qin J. H. (2018). A Microfluidic Strategy for Controllable Generation of Water-In-Water Droplets as Biocompatible Microcarriers. Small 14 (36), e1801095. 10.1002/smll.201801095 30091845

[B27] Lopa S. Piraino F. Talò G. Mainardi V. L. Bersini S. Pierro M. (2020). Microfluidic Biofabrication of 3D Multicellular Spheroids by Modulation of Non-geometrical Parameters. Front. Bioeng. Biotechnol. 8, 366. 10.3389/fbioe.2020.00366 32432090 PMC7214796

[B28] Ma T. Gao X. Dong H. He H. Cao X. (2017). High-throughput Generation of Hyaluronic Acid Microgels via Microfluidics-Assisted Enzymatic Crosslinking And/or Diels-Alder Click Chemistry for Cell Encapsulation and Delivery. Appl. Mater. Today 9, 49–59. 10.1016/j.apmt.2017.01.007

[B29] Mao A. S. Shin J.-W. Utech S. Wang H. Uzun O. Li W. (2017). Deterministic Encapsulation of Single Cells in Thin Tunable Microgels for Niche Modelling and Therapeutic Delivery. Nat. Mater 16 (2), 236–243. 10.1038/nmat4781 27798621 PMC5372217

[B30] Mohamed M. G. A. Ambhorkar P. Samanipour R. Yang A. Ghafoor A. Kim K. (2020). Microfluidics-based Fabrication of Cell-Laden Microgels. Biomicrofluidics 14 (2), 021501. 10.1063/1.5134060 32161630 PMC7058428

[B31] Mohamed M. G. A. Kheiri S. Islam S. Kumar H. Yang A. Kim K. (2019). An Integrated Microfluidic Flow-Focusing Platform for On-Chip Fabrication and Filtration of Cell-Laden Microgels. Lab. Chip 19 (9), 1621–1632. 10.1039/c9lc00073a 30896015

[B32] Moon B.-U. Jones S. G. Hwang D. K. Tsai S. S. H. (2015). Microfluidic Generation of Aqueous Two-phase System (ATPS) Droplets by Controlled Pulsating Inlet Pressures. Lab. Chip 15 (11), 2437–2444. 10.1039/c5lc00217f 25906146

[B33] Najah M. Griffiths A. D. Ryckelynck M. (2012). Teaching Single-Cell Digital Analysis Using Droplet-Based Microfluidics. Anal. Chem. 84 (3), 1202–1209. 10.1021/ac202645m 22229495

[B34] Roper S. J. Linke F. Scotting P. J. Coyle B. (2021). 3D Spheroid Models of Paediatric SHH Medulloblastoma Mimic Tumour Biology, Drug Response and Metastatic Dissemination. Sci. Rep-uk 11 (1). 10.1038/s41598-021-83809-6 PMC7895940 33608621

[B35] Shao F. Yu L. Zhang Y. An C. Zhang H. Zhang Y. (2020). Microfluidic Encapsulation of Single Cells by Alginate Microgels Using a Trigger-Gellified Strategy. Front. Bioeng. Biotechnol. 8, 583065. 10.3389/fbioe.2020.583065 33154965 PMC7591722

[B36] Shembekar N. Hu H. Eustace D. Merten C. A. (2018). Single-Cell Droplet Microfluidic Screening for Antibodies Specifically Binding to Target Cells. Cel Rep. 22 (8), 2206–2215. 10.1016/j.celrep.2018.01.071 PMC5842027 29466744

[B37] Siltanen C. Yaghoobi M. Haque A. You J. Lowen J. Soleimani M. (2016). Microfluidic Fabrication of Bioactive Microgels for Rapid Formation and Enhanced Differentiation of Stem Cell Spheroids. Acta Biomater. 34, 125–132. 10.1016/j.actbio.2016.01.012 26774761 PMC4811722

[B38] Sivakumar H. Devarasetty M. Kram D. E. Strowd R. E. Skardal A. (2020). Multi-Cell Type Glioblastoma Tumor Spheroids for Evaluating Sub-population-specific Drug Response. Front. Bioeng. Biotechnol. 8, 538663. 10.3389/fbioe.2020.538663 33042963 PMC7523412

[B39] Tao T. Wang Y. Chen W. Li Z. Su W. Guo Y. (2019). Engineering Human Islet Organoids from iPSCs Using an Organ-On-Chip Platform. Lab. Chip 19 (6), 948–958. 10.1039/c8lc01298a 30719525

[B40] Tekin H. Tsinman T. Sanchez J. G. Jones B. J. Camci-Unal G. Nichol J. W. (2011). Responsive Micromolds for Sequential Patterning of Hydrogel Microstructures. J. Am. Chem. Soc. 133 (33), 12944–12947. 10.1021/ja204266a 21766872 PMC3206098

[B41] Türker E. Demirçak N. Arslan-Yildiz A. (2018). Scaffold-free Three-Dimensional Cell Culturing Using Magnetic Levitation. Biomater. Sci. 6 (7), 1745–1753. 10.1039/c8bm00122g 29700506

[B42] Vadivelu R. K. Kamble H. Shiddiky M. J. A. Nguyen N.-T. (2017). Microfluidic Technology for the Generation of Cell Spheroids and Their Applications. Micromachines-Basel 8 (4). 10.3390/mi8040094

[B43] Zhang W. J. Li D. H. Jiang S. W. Galan E. A. Jiang S. W., (2021). Microfluidic Droplets as Structural Templates for Matrigel to Enable 1-week Large Organoid Modeling. Chem. Eng. Sci. 238. 10.1016/j.ces.2021.116632

[B44] Zhu K. Yu Y. Cheng Y. Tian C. Zhao G. Zhao Y. (2019). All-Aqueous-Phase Microfluidics for Cell Encapsulation. ACS Appl. Mater. Inter. 11 (5), 4826–4832. 10.1021/acsami.8b19234 30648845

