# Supplementary material for: One-Step Generation and Purification of Cell-Encapsulated Hydrogel Microsphere With an Easily Assembled Microfluidic Device
Source: Front Bioeng Biotechnol. 2022 Jan 28;9:816089. doi: 10.3389/fbioe.2021.816089 (PMC8831896; doi:10.3389/fbioe.2021.816089)
Supplement: Supplementary file 1 [file DataSheet1.docx]

Supplementary Material

# Materials and Methods

**1.1 Cell culture**

The colon cancer cell line HCT116 and the glioma cancer cell line U87 were purchased from the American Type Culture Collection (ATCC, Rockville, MD), and they were cultured in Dulbecco’s Modified Eagle Medium (DMEM; GIBCO) supplemented with 10% fetal bovine serum (FBS; GIBCO) and 100 U/mL penicillin/streptomycin (P/S; GIBCO) in T25 cell culture flasks (Thermo Scientific). Cells were cultured at 37 °C in a humidified 5% CO_2_ and 95% air atmosphere.

# Supplementary Figures and Tables


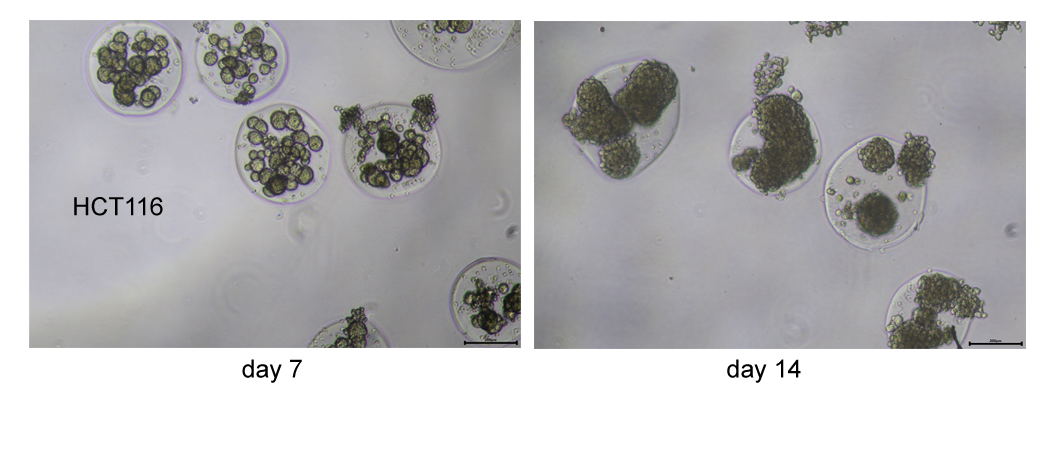


**Supplementary Figure 1** Microscopy image of HCT116-laden hydrogel microspheres after culturing for 7 and 14 days. The scale bar represents 200 µm.


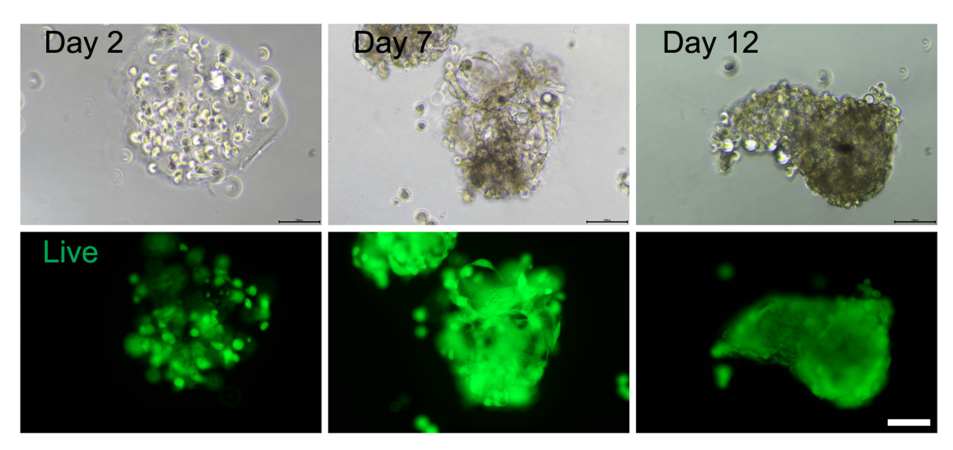


**Supplementary Figure 2** Fluorescence microscopy image of U87-laden hydrogel microspheres after culturing for 2, 7, and 12 days. The scale bar represents 100 µm.


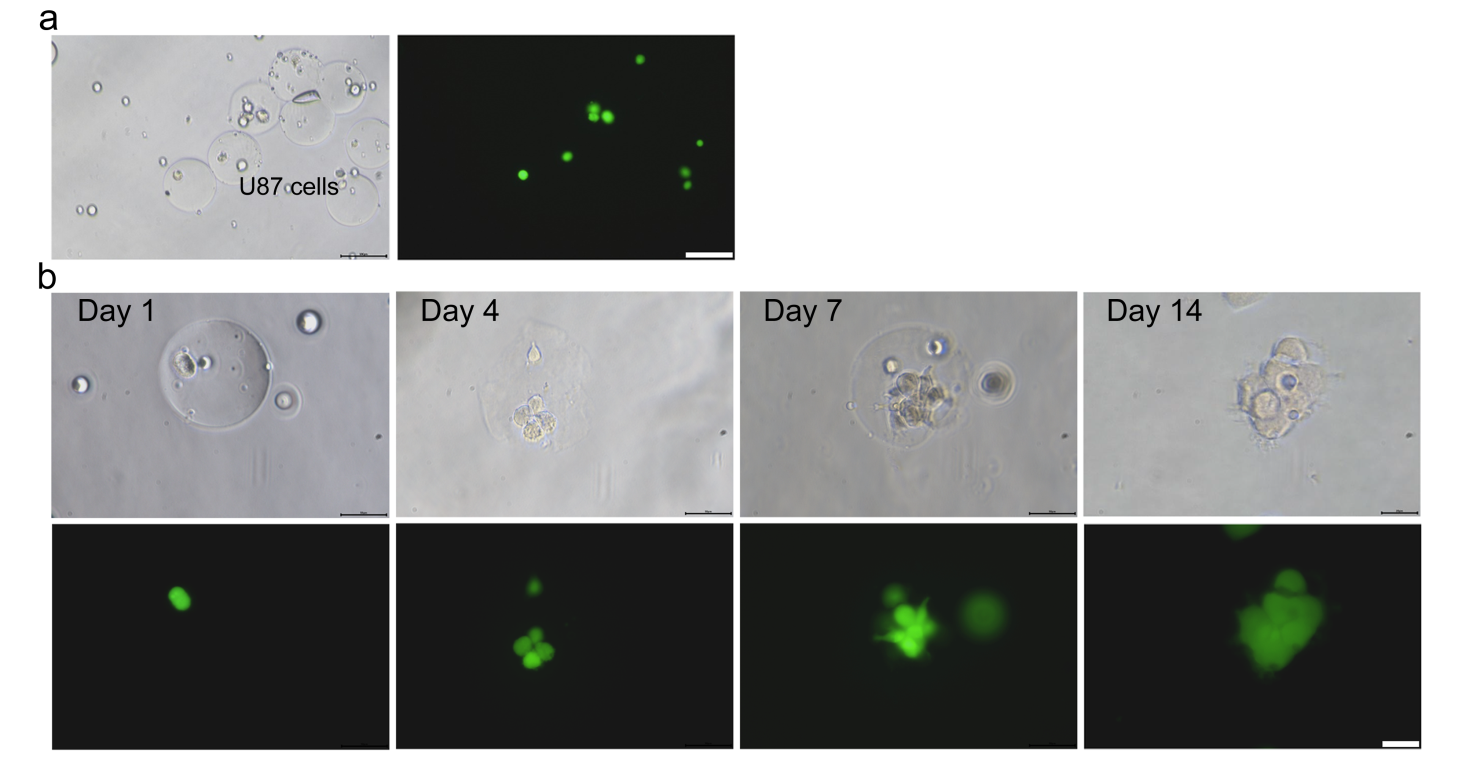


**Supplementary Figure 3** Generation and culture of single cell-laden hydrogel microspheres. (a) Representative image of U87 cell-laden hydrogel microspheres. The scale bar represents 100 µm. （b）Microscopy image of U87-laden hydrogel microspheres after culturing for 1, 4, 7 and 14 days. The scale bar represents 50 µm.
